# Machine Learning-Based Identification of Colon Cancer Candidate Diagnostics Genes

**DOI:** 10.3390/biology11030365

**Published:** 2022-02-25

**Authors:** Saraswati Koppad, Annappa Basava, Katrina Nash, Georgios V. Gkoutos, Animesh Acharjee

**Affiliations:** 1 Department of Computer Science and Engineering, National Institute of Technology Karnataka, Mangalore 575025, India; saraswatikoppad@gmail.com (S.K.); annappa@ieee.org (A.B.); 2 College of Medical and Dental Sciences, University of Birmingham, Birmingham B15 2TT, UK; katrinanash649@outlook.com; 3 Institute of Cancer and Genomic Sciences, University of Birmingham, Birmingham B15 2TT, UK; g.gkoutos@bham.ac.uk; 4 Institute of Translational Medicine, University of Birmingham, Birmingham B15 2TT, UK; 5 NIHR Surgical Reconstruction and Microbiology Research Centre, University Hospital Birmingham, Birmingham B15 2WB, UK; 6 MRC Health Data Research UK (HDR UK), Midlands Site, Birmingham B15 2TT, UK; 7 NIHR Experimental Cancer Medicine Centre, Birmingham B15 2TT, UK; 8 NIHR Biomedical Research Centre, University Hospital Birmingham, Birmingham B15 2TT, UK

**Keywords:** biomarker identification, transcriptomics, machine learning, prediction, variable selection

## Abstract

**Simple Summary:**

We developed a predictive approach using different machine learning methods to identify a number of genes that can potentially serve as novel diagnostic colon cancer biomarkers.

**Abstract:**

Background: Colorectal cancer (CRC) is the third leading cause of cancer-related death and the fourth most commonly diagnosed cancer worldwide. Due to a lack of diagnostic biomarkers and understanding of the underlying molecular mechanisms, CRC’s mortality rate continues to grow. CRC occurrence and progression are dynamic processes. The expression levels of specific molecules vary at various stages of CRC, rendering its early detection and diagnosis challenging and the need for identifying accurate and meaningful CRC biomarkers more pressing. The advances in high-throughput sequencing technologies have been used to explore novel gene expression, targeted treatments, and colon cancer pathogenesis. Such approaches are routinely being applied and result in large datasets whose analysis is increasingly becoming dependent on machine learning (ML) algorithms that have been demonstrated to be computationally efficient platforms for the identification of variables across such high-dimensional datasets. Methods: We developed a novel ML-based experimental design to study CRC gene associations. Six different machine learning methods were employed as classifiers to identify genes that can be used as diagnostics for CRC using gene expression and clinical datasets. The accuracy, sensitivity, specificity, F1 score, and area under receiver operating characteristic (AUROC) curve were derived to explore the differentially expressed genes (DEGs) for CRC diagnosis. Gene ontology enrichment analyses of these DEGs were performed and predicted gene signatures were linked with miRNAs. Results: We evaluated six machine learning classification methods (Adaboost, ExtraTrees, logistic regression, naïve Bayes classifier, random forest, and XGBoost) across different combinations of training and test datasets over GEO datasets. The accuracy and the AUROC of each combination of training and test data with different algorithms were used as comparison metrics. Random forest (RF) models consistently performed better than other models. In total, 34 genes were identified and used for pathway and gene set enrichment analysis. Further mapping of the 34 genes with miRNA identified interesting miRNA hubs genes. Conclusions: We identified 34 genes with high accuracy that can be used as a diagnostics panel for CRC.

## 1. Introduction

Colorectal cancer (CRC) is the third most common cause of death due to cancer and the fourth most commonly diagnosed cancer worldwide [1,2]. Considering demographic estimates, nearly 2.2 million new cases and about 1.1 million deaths are expected by 2030, and the global burden of CRC is estimated to increase by 60% [3]. CRC cancer is a genotype and phenotype heterogeneous disease, characterized by a display of distinct molecular signatures [4]. Around 1.4 million new cases and nearly 700,000 deaths were recorded in 2012 due to colorectal cancer [5].

Advancements in omics technologies, such as microarrays, RNAseq [6], next-generation sequencing (NGS) [7], and mass spectrometry [8], have enabled employing molecular markers for the diagnosis of CRC [9]. For example, recent studies have used gene microarrays, as well as high-throughput sequencing technologies, to explore differential expressing novel genes in colon cancer [10]. Fang-Ze et al. [11] reported that *CLCA1* may be a candidate diagnostic and prognostic differentially expressed gene or biomarker for colon cancer. Li et al. [12] identified *CDK1* and *CDC20* genes as candidate targets for diagnosis of CRC. Most studies reported individual markers such as the *CEA*, *CK19*, and *CK20* genes [13]. However, the resulting specificity (89%) and sensitivity (78%) of those biomarkers have rendered them unsuitable for the development of a noninvasive diagnostic method for the detection of colon cancer [14]. Dasi et al. [15] and Schiedeck et al. [16] investigated *TERT*, *GCC*, *MAGEA*, *TS*, *CGM2*, and *L6* as biomarkers for detecting colon cancer, reporting a sensitivity and specificity of around 85% and 95%. Furthermore, Liu et al. [17] identified seven prognostic genes, namely, *TIMP1*, *LZTS3*, *AXIN2*, *CXCL1*, *ITLN1*, *CPT2*, and *CLDN23*, for the application of novel diagnostic and prognostic biomarkers for the treatment of colon cancer.

Torres et al. [18] investigated the proteome profiling of human and mouse tissue which revealed a novel association of cancer-associated fibroblasts with cancer progression. This study further unveiled the role of the *LTBP2*, *CDH11*, *OLFML3*, *CALU*, *CDH11*, and *FSTL1* proteins in migration and invasion of CRC and, hence, their use as a biomarker. Moreover, Kim et al. (2019) [19] identified abnormal concentrations of the taurine, alanine, 3-aminoisobutyrate, and citrate metabolites from urine samples in CRC patients. 

Although the various molecular characteristics, biological markers, and therapeutic targets of colon cancer previously discovered have contributed significantly to its diagnosis and treatment, the biological complexity, outcome severity, and high metastasis of this complex disease necessitate further predictive and prognostic biomarker identification [20,21]. Currently, CRC prognosis is based on a classification of clinicopathological features, including, tumor, node, metastasis (TNM) stage, cancer numbers, histologic type (mucinous carcinoma or signet ring-cell carcinoma), histology type, tumor grade, tumor size, number of lymph nodes, and tumor location [22]. Furthermore, the right and left localization and the excision of lymph nodes are included in the histological type and grading in the prognosis of colorectal cancer [23]. 

This study aimed to design and develop novel ML-based, computationally efficient platforms to study CRC gene associations and identify signature genes used as diagnostics markers across transcriptomics datasets.

## 2. Methods

In this study, we used three gene expression datasets (GSE44861, GSE20916, GSE113513), available from the GEO database [24], and applied six different machine learning methods (Adaboost, ExtraTrees, logistic regression, naïve Bayes, random forest, and XGBoost) to identify genes that can be used as diagnostics markers. We used different combinations of the GSE44861, GSE20916, and GSE113513 datasets for training and validation. We then performed an enrichment analysis and associated the resulting gene signatures with miRNA. Lastly, we estimated the number of samples required for the markers selected for the future validation experiments. 

### 2.1. Data

**Figure 1:**
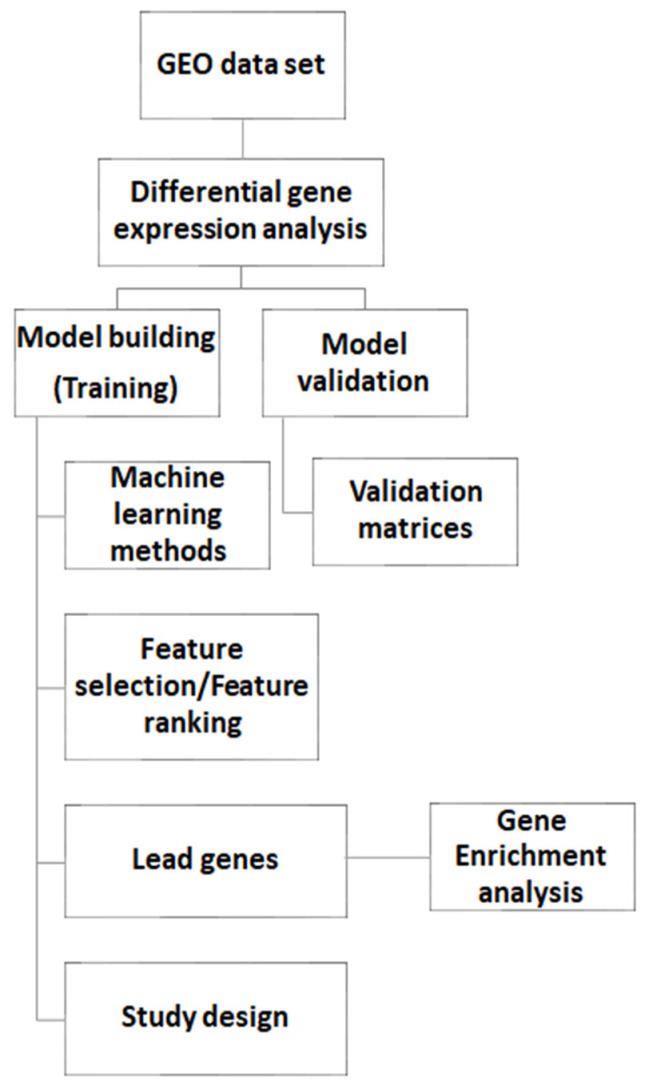
A schematic representation of the biomarker identification workflow.

The gene expression matrixes and clinical data were downloaded from the GEO database repository (https://www.ncbi.nlm.nih.gov/geo/) accessed on 1 October 2020. The details of the datasets used in this study are summarized in Table 1. The detailed workflow of the methods and process used in this study is presented in Figure 1.

#### Differentially Expressed Genes (DEGs) Identified by GEO2R

GEO2R (http://www.ncbi.nlm.nih.gov/geo/geo2r, accessed on 5 January 2021), an online data analysis tool, was used to identify differentially expressed genes (DEGs) between colon cancer patients and healthy controls. We used three GEO series, namely GSE20916, GSE44861, and GSE113513, and identified differential expressed genes. Genes without a corresponding gene symbol and genes with more than one probe set were removed. Adjusted *p*-values ≤ 0.0001 were considered statistically significant. Subsequently, the top 500 most statistically significant DEG genes from each dataset were selected for further analysis. 

### 2.2. Machine Learning Algorithms and Predictive Analytics

Six different machine learning algorithms, namely, Adaboost [28], ExtraTrees [29], logistic regression [30,31], naïve Bayes (NB) classifier [32], random forest [33], and XGBoost [34], were employed to develop models using the selected GEO datasets (GSE44861, GSE20916, and GSE113513). These datasets were employed to generate different combinations of training and test data to assess the derived models’ performance.

The python Scikit-learn libraries [35] were employed for the implementation of the different classifiers and feature selection methods. 

#### 2.2.1. Hyperparameter Optimization

We used the GridSearchCV [35] function to find the optimal values for each model hyperparameter. GridSearchCV is a function, available as part of the Scikit-learn’s library, that caters the looping through predefined hyperparameters and the fitting of the model on the training set. GridSearchCV uses all the combinations of the predefined parameter values and evaluates a model’s performance for each combination using cross-validation. The accuracy results obtained for every hyperparameter combination can then be used to identify the best-performing model.

#### 2.2.2. Machine Learning Model Evaluation

The analysis was carried out using three different GEO datasets (GSE44861, GSE20916, and GSE113513) as training and testing data for performance comparison in a combinatorial way with six different machine learning models including logistic regression [36], naïve Bayes [37], random forest [38], ExtraTrees [39], Adaboost [40], and XGBoost [41]. Each model was evaluated with different evaluation metrics such as precision, recall [42], specificity, sensitivity [43], F1 score, AUROC [44], and accuracy.

We also included multiple validation strategies to validate the performance of the model. The most commonly used k-fold cross-validation technique was applied in our experimental work. In the k-fold (here, k = 5) cross-validation technique, the dataset is randomly split into k subsets, whereby k − 1 subsets are used for training, and the remaining subset is used for testing; the is process repeated k times. In addition to this, we used resampling with the bootstrap method and leave-one-out cross-validation (LOOCV) in our experimental work for validation of the model performance. The model performance was evaluated for the mean value of performance metrics over 100 iterations. In the LOOCV method, the dataset is split into training data considering all data samples, excluding one data sample used as the test dataset. The model developed with training data finally measures the mean performance value for the repeated process. The experimental results in this method for different models are also provided in Supplementary Table S1.

#### 2.2.3. Feature Selection

We performed feature selection using two methods, mean decrease in impurity (MDI) [45] and Boruta [46], for the selection of important genes. MDI or Gini importance [47] computes the total reduction in loss or impurity contributed by all splits for a given feature. This method evaluates the importance of a variable Xm for predicting Y by adding up the weighted impurity decreases p(t) ∆i(st,t) for all nodes t where Xm is used, averaged over all NT trees in the forest as shown in the equation below.

$$\mathrm{Imp}\left(X_{m}\right)=\frac{1}{N_{T}}\underset{T}{\sum }\overset{}{\underset{t\in T_{\mathrm{iv}\left(s_{t}\right)}=X_{m}}{\sum }}\;p\left(t\right)\Delta i\left(s_{t},t\right),$$

where p(t) is the proportion Nt/N of samples reaching t, and v(st) is the variable used in split st. When using the Gini index as an impurity function, this measure is known as the Gini importance or mean decrease Gini. MDI is computationally very efficient and has been widely used in a variety of applications. Gini importance represents the total decrease in node impurity, i.e., how much the model fit or accuracy decreases when dropping a variable. A larger decrease in node impurity results in a more significant variable. The top 15 genes across 10 iterations were selected with the MDI technique.

In addition to MDI, we also used Boruta which is a feature selection algorithm and works as a wrapper algorithm around random forest [48]. It attempts to capture all the important, interesting features from a dataset with respect to an outcome variable and can be used in combination with tree-based ensemble learning algorithms.

### 2.3. Gene Enrichment Analysis

Gene ontology (GO) enrichment analysis of DEGs was carried out using the FunRich (functional enrichment analysis tool) (http://www.funrich.org/, accessed on 25 January 2021). DEGs were classified according to the biological process and cellular component GO collections. Biological terms with an FDR *p*-value lower than 0.05 were considered significantly enriched. Correction for multiple hypothesis testing was carried out by the Benjamini–Hochberg method.

#### 2.3.1. Association of the Gene Markers with miRNA

We used the NetworkAnalyst (www.networkanalyst.ca, accessed on 28 January 2021) [49] tool and more specifically the gene–miRNA module that employs the miRTarBase v8 database to calculate the number of the connections or links for each gene, also termed degrees.

#### 2.3.2. Sample Size Estimates for Future Validation Experiments

We then used PowerTools (https://joelarkman.shinyapps.io/PowerTools/, accessed on 10 February 2021) [50] to estimate the number of samples required for future experiments.

## 3. Results

### 3.1. Differential Expressed Genes (DEGs)

We identified the top 500 DEGS across each of the GEO datasets examined. For the GSE44861 dataset, 324 genes were found to be upregulated and 176 genes were downregulated, while, for the GSE20916 and the GSE113513 datasets, 171 and 223 genes were upregulated and 329 and 277 genes were downregulated, respectively. The identified differentially expressed genes and their respective *p*-values, as well as the fold changes, are listed in Supplementary Table S2.

#### Performance Evaluation

For each of the three GEO datasets examined, their respective DEGs were used as features across six different classification models, namely, Adaboost, ExtraTrees, logistic regression, naïve Bayes classifier, random forest, and XGBoost. The performance of these models was evaluated against different combination of training and test datasets.

The results of the different performance metrics for each classifier are presented in Supplementary Table S1. With GSE44861 as training data and GSE20916 as test data, the random forest model achieved better performance with an accuracy of 98.2% and 90% using the bootstrap and LOOCV methods, respectively. With GSE44861 as training and GSE113513 as testing data, the logistic regression model achieved an accuracy of 96.4% and 84% using bootstrap and LOOCV, respectively. When we used GSE20916 as training data and GSE44861 as testing data, the naïve Bayes classifier achieved an accuracy of 90.1% and 96% using bootstrap and LOOCV, respectively. With GSE20916 as training data and GSE113513 as testing data, logistic regression resulted in better performance. With GSE113513 as training and GSE44861 as testing data, the ExtraTree classifier model achieved better performance. With GSE113513 as training data and GSE20916 as testing data, none of the models achieved good performance.

**Figure 2:**
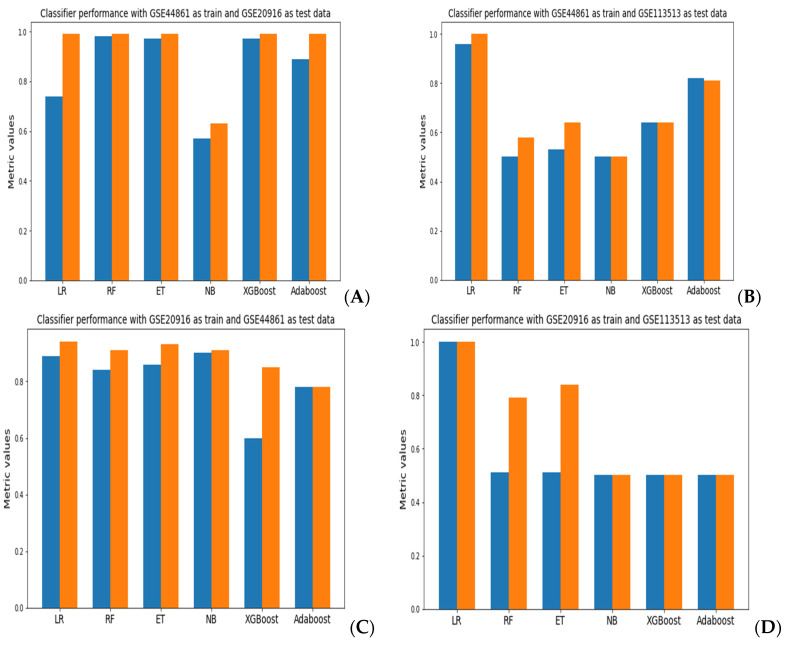

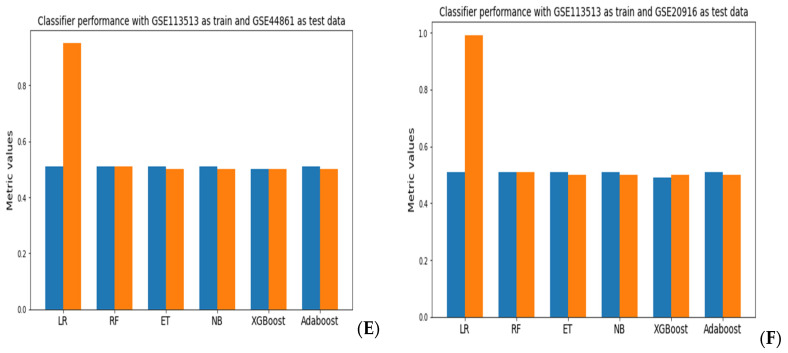
A comparison of accuracy (blue) and AUROC (orange) values obtained across the different classifiers using combinations of the GEO datasets as training and test datasets. (**A**) GSE44861 (training) and GSE20916 (test); (**B**) GSE44861 (training) and GSE20916 (test); (**C**) GSE20916 (training) and GSE44861 (test); (**D**) GSE20916 (training) and GSE113513 (test); (**E**) GSE113513 (training) and GSE44861 (test); (**F**) GSE113513 (training) and GSE20916 (test).

A comparison of the accuracy and AUROC results for each model evaluations is presented in Figure 2. When using GSE44861 as training data and GSE20916 as test data, the random forest classifier achieved the best performance across all classifiers with an accuracy of 98.2% and an AUROC of 99.9% (Figure 2A). With GSE44861 as training data and GSE113513 as test data, a logistic regression model achieved an accuracy of 96.4% and an AUROC of 99% (Figure 2B). When using GSE20916 as training data and GSE44861 as test data, the naïve Bayes classifier exhibited the best performance with an accuracy of 90.1% and AUROC of 90%, as shown in Figure 2C. Using GSE20916 as the training data and GSE113513 as the test data, the logistic regression model achieved the best performance (Figure 2D). Lastly, with GSE113513 as the training data and GSE44861 as the test data, as well as with GSE113513 as the training data and GSE20916 as the test data, all classifiers achieved an accuracy of 50% to 51% and an AUROC of 50% to 51%, apart from logistic regression, which resulted in an AUROC of 99% (Figure 2E,F).

**Figure 3:**
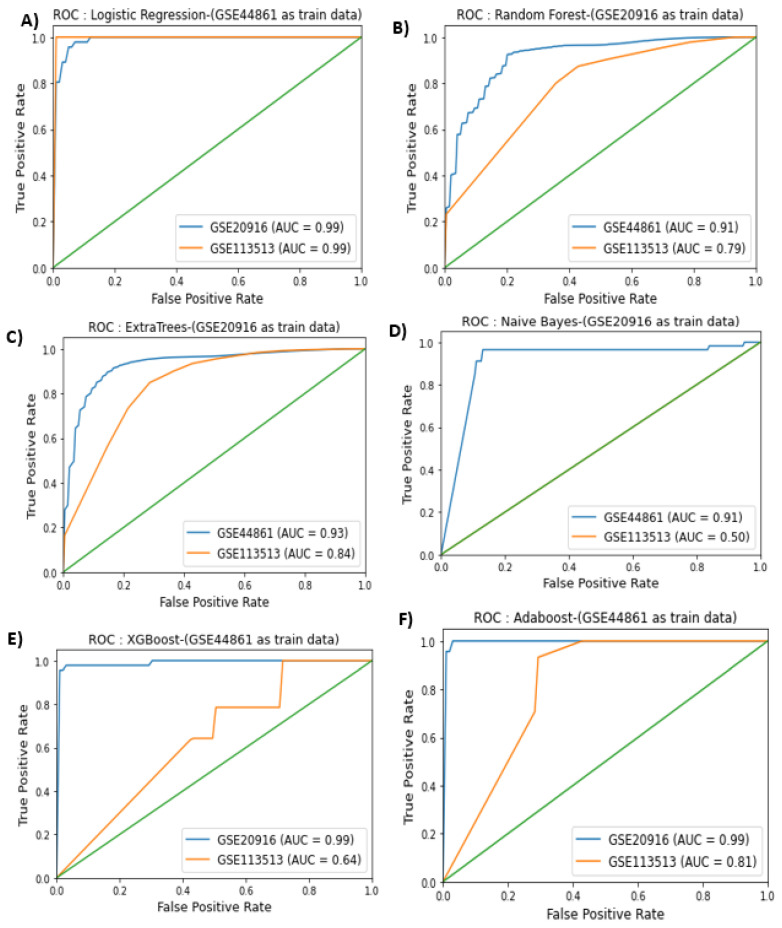
ROC curves for the different classifiers. (**A**) Performance of logistic regression model with GSE44861 as training and GSE20916, GSE113513 as test data; (**B**) performance of random forest model with GSE20916 as training and GSE44861, GSE113513 as test data; (**C**) performance of ExtraTrees model with GSE20916 as training and GSE44861, GSE113513 as test data; (**D**) performance of naïve Bayes model with GSE20916 as training and GSE44861, GSE113513 as test data; (**E**) performance of XGBoost model with GSE44861 as training and GSE20916, GSE113513 as test data; (**F**) performance of Adaboost model with GSE44861 as training and GSE20916, GSE113513 as test data.

The AUROC plots for the models that had the best performance across the different training and test data combinations are presented in Figure 3. Across the three datasets tested, random forest and logistic regression achieved the best performance when we combined GSE44861 and GSE20916 datasets as training and test data. However, none of the classifiers assessed achieved a good performance using the GSE113513 dataset. The best performances of each classification model are represented as AUROC plots. Overall, the random forest models exhibited consistently better performance across all classification models tested.

### 3.2. Gene Selection

**Figure 4:**
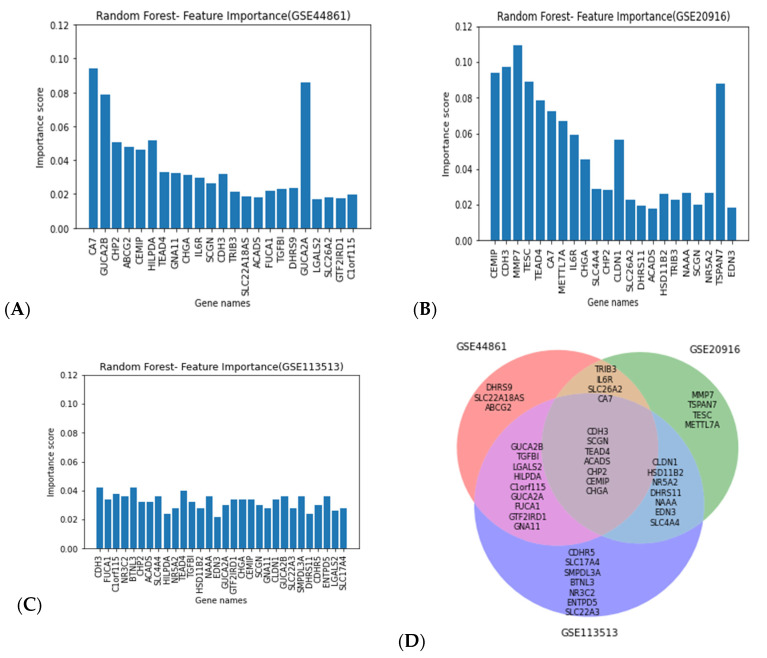
Important genes selected using the mean decrease in impurity (MDI) technique in combination with random forest classifier. The *x*-axis represents the gene names, and the *y*-axis represents importance score values across the GSE44861 (**A**), GSE20916 (**B**), and GSE113513 datasets (**C**). The common genes from all three datasets (**D**).

Random forest classification, on the basis of the performance previously reported, was applied in combination with MDI to select the top 15 genes with the highest importance score in 10 different iterations. We then identified the union of all the genes selected from all 10 iterations. Figure 4 shows the important genes selected using the mean decrease in impurity (MDI) technique in combination with the random forest classifier. Figure 4A depicts the important genes selected using the GSE44861 dataset, while Figure 4B presents the important genes selected using the GSE20916 dataset.

#### Gene Ontology (GO) Enrichment Analysis

**Figure 5:**
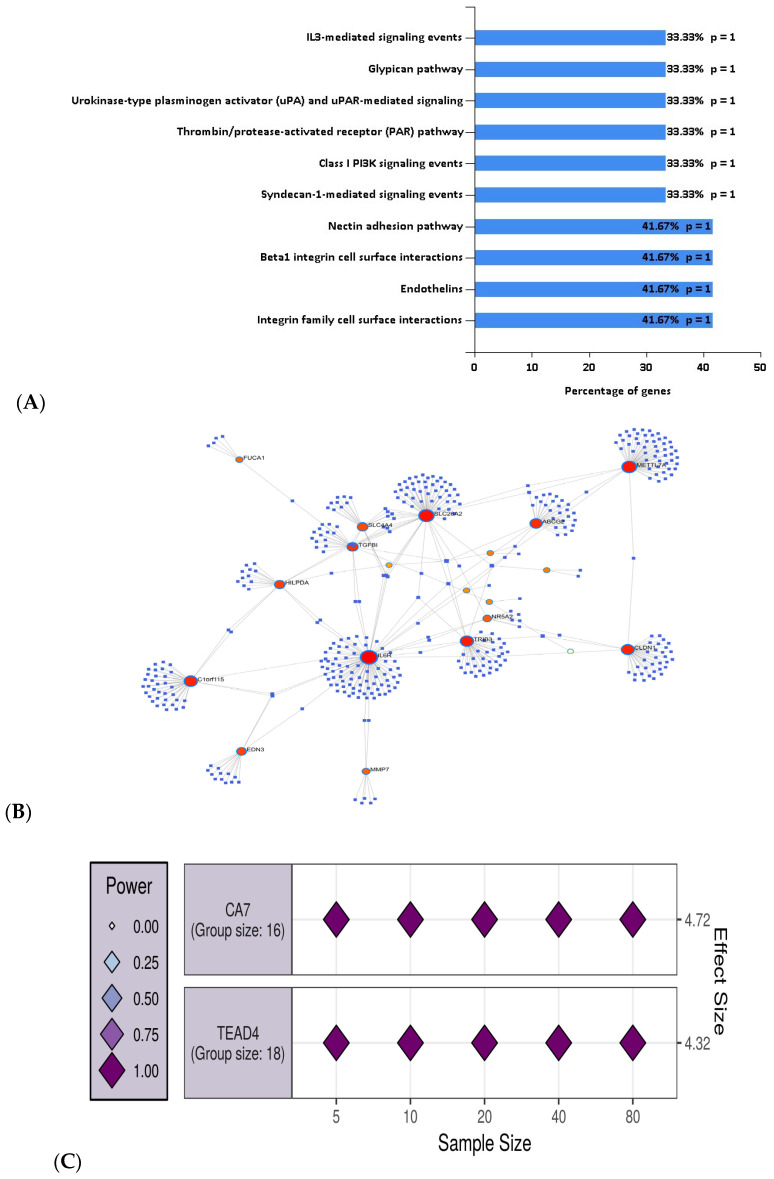
(**A**) Pathway enrichment analysis with the genes selected using the MDI method; (**B**) mapping of the 19 most interacting genes out of 34 genes with miRNAs and their interconnections; (**C**) the two clusters of genes with representative genes *CA7* and *TEAD4* for the GSE44861 are visualized by the largest effect size. The effect size of each assessed variable is shown along the *y*-axis, with a series of sample sizes along the *x*-axis.

MDI in combination with the random forest classifier for feature selection resulted in the selection of 34 genes that were used for the pathway and gene set enrichment analysis. These genes were found to be associated with a number of molecular functions including cell adhesion molecule activity (*CDH3* and *CLDN*), transporter activity (*ABCG2, SLC22A18AS*, and *SLC26A2*), catalytic activity (*CA7, DHRS9*, and *HSD11B2*) and oxidoreductase activity (*ACADS* and *DHRS11*). The pathways for which 34 genes were found to be enriched are presented in Figure 5A.

### 3.3. Associating Selected Genes with miRNA Using NetworkAnalyst

We mapped the 34 identified genes using the NetworkAnalyst tool and found that 19 genes out of 34 genes formed hub genes (Figure 5B). For example, *IL6R* had the highest number of miRNA interactions (degree, 94). A list of the identified genes and their miRNA associations is provided in the Supplementary Table S3.

Lastly, we also performed a power analysis over the GSE44861 dataset. For this purpose, we used the 34 genes that were identified and ranked by the random forest algorithm. We then applied hierarchical clustering over these 34 genes and identified two clusters. We selected the genes that presented the highest correlation across normal vs. cancer samples.

*CA7* and *TEAD4* were selected as representative genes across the two clusters as they had the highest correlation with the normal vs. CRC samples (i.e., lowest *p*-values). For both clusters of genes including *CA7* and *TEAD4*, we estimated *N* = 5 samples, required for both control and CRC samples. Figure 5C represents the number of the estimated samples required for genes from each cluster.

## 4. Discussion

The three GEO datasets used in our experimental work with six different machine learning methods were validated across different combinations of training and test datasets. The performance of each model was reported and compared using a number of performance metrics, such as accuracy, sensitivity, specificity, AUC, etc. The random forest method showed the best performance against the GSE44861 and GSE20916 datasets when used as a combination of training and test data. It was less prone to overfitting when compared to the other methods used. This method has also been applied successfully in other diseases such as NAFLD [51], obesity [52], and IBD [53]; therefore, we applied the random forest method to select the important features from these two datasets.

The GSE113513 dataset had a lower number of samples or observations compared to the GSE44861 and GSE20916 datasets, which resulted in lower performance compared to the other datasets, thus indicating an overfitting problem. We used multiple approaches to protect against the overfitting problem, such as the widely used fivefold cross-validation, LOOCV, and bootstrapping. Compared with k-fold cross validation and LOOCV, the bootstrap method could use the entire sample in model development and validation, thus helping to estimate optimism and measure overfitting. The optimism-corrected estimated performance by the bootstrap method is relatively stable because it uses the full sample size and the bootstrap samples vary in composition [54]. We incorporated 100 iterations with the bootstrap method for the experimental work, and each of these evaluation metrics were averaged over these 100 iterations. Datasets GSE44861 and GSE20961 were observed to perform better, and the random forest method was chosen for the feature selection process.

The gene ontology enrichment analysis identified several genes and their associated pathways, most notably, cell adhesion molecule activity, transporter activity, catalytic activity, and oxidoreductase activity. *CDH3*, a gene encoding P-cadherin that forms a major component of the adherens junctions that are essential for cell adhesion, has been identified as being upregulated in CRC in multiple studies and as a diagnostic or prognostic marker [55,56]. Conversely, *CLDN*, encoding for the claudin protein forming tight junctions, has been found to be a potential diagnostic marker with downregulation in CRC patients [56,57]. Furthermore, previous research has postulated that the *HDS11B2* gene, involved in catalytic activity pathways, plays a vital role in migration, invasion, and metastasis of CRC [58]. Other genes identified to be involved in catalytic activity (*CA7* and *DHRS9*) have been found to be downregulated in CRC cells, and have been proposed as promising diagnostic and/or prognostic markers [59,60]. Genes associated with transporter activity have also been identified in existing studies. Of particular note is the upregulation of the *ABCG2* gene, which has been postulated to play a protective role against oxidative stress through cell signaling pathways, which may explain why it has been found to be upregulated in CRC [61,62,63]. Similarly, genes involved in oxidoreductase activity (*ACADS* and *DHRS11*) have been found to be downregulated in previous studies [64,65]. These genes are involved in fatty-acid metabolism and energy production within mitochondria; thus, their downregulation may partially explain the changes in metabolism often observed in cancer cells [66]. Many of the identified genes have been previously associated with colon cancer via miRNA interactions. Multiple studies, including Bian et al., Hua et al., and Xu et al., have reported that serum *IL-6* may be a potential biomarker for CRC diagnosis and a miR-34a target [67]. IL6R has also been implicated in other cancer types, including prostate cancer [68]. Another gene, *SLC4A4*, was found to be significantly correlated with shorter survival of CRC patients and a marker of poorer progression for patients with breast cancer, lung cancer, gastric cancer, and ovarian cancer. This suggests a potential role *of SLC4A4* in tumor suppression, as well as in prognostic prediction in multiple malignancies, including CRC, thus representing a potential novel therapeutic CRC target [69]. Yang et al. (2019) [70] identified a similar *SLC4A4* expression association and proposed the expression of six further genes, namely, *SGCG, CLDN23, CCDC78, SLC17A7, OTOP3,* and *SMPDL3A*, as novel colon cancer prognostic biomarkers. Zhang et al. (2020) [71] reported that hsa_circRNA_001587 upregulates *SLC4A4* expression to inhibit migration, invasion, and angiogenesis of pancreatic cancer cells via binding to microRNA-223. Furthermore, Mencia et al. (2011) reported miR-224 to be one of the most differentially expressed miRNAs associated with *SLC4A4* [72]. Andersen et al. (2015) [73] reported changes in gene expression levels (high *ABCC2* and low *ABCG2*) as early events in the colon adenoma–carcinoma sequence. Moreover, miR-132 has been reported to regulate the *SIRT1/CREB/ABCG2* signaling pathway, contributing to cisplatin resistance and serving as a novel therapeutic target against gastric cancer [74]. Cherradi et al. found *CLDN1* to be significantly overexpressed (*p* < 0.001) in CRC samples, and they proposed it as a new potential therapeutic target of miR-7-2 [75]. Lastly, Miwa et al. (2011) [76] reported *CLDN1* as a target of TCF/LEF signaling, while Singh et al. (2011) [77] suggested the involvement of *CLDN1* in the regulation of the WNT signaling pathway.

Our approach utilized a limited number of public datasets, and the potential causal relationships identified necessitate experimental validation. We did not consider the effect of multiple factors, such as age, gender, ethnicity, and tumor grade and stage, on gene expression patterns since we focused only on genes that have been previously reported as having significant variation between control and cancer samples. In the context of translational medicine [78], further research is required to investigate the selected prognostic/diagnostic signature’s clinical utility in predicting clinical outcomes in various tumor types.

In CRC diagnostics, colonoscopy is the current gold-standard screening method. However, this approach has some limitations that include internal hemorrhage, colonic perforation, and cardiorespiratory problems [79].

Another approach is the guaiac fecal occult blood test (gFOBT) [80], which detects hemoglobin peroxidase activity in the feces, and it is the most often used noninvasive screening procedure. Although FOBT is a simple and inexpensive way to screen for CRC, it has a high percentage of false positives and false negatives.

As a result, alternative CRC screening approaches that are cost-effective, noninvasive, easily quantifiable, and accurate are urgently needed. Thus, gene signature-based biomarkers in the clinical applications in CRC are required for early cancer detection, prognostic stratification, and surveillance [80]. Genes identified in this study will need to go through targeted validation experiments using qPCR. A new trial needs to be set up to replicate the gene signature’s effect. This step will ensure the clinical efficacy of those markers identified and will allow a better clinical decision on CRC [81].

## 5. Conclusions

This study aimed to identify novel genes associations with CRC that can potentially be used as diagnostic markers in translational research. To achieve this, we applied a predictive analytics approach that employed a variety of machine learning methods. In addition, we estimated the required number of samples for future validation experiments.

## Figures and Tables

**Table 1:** List of the datasets and platforms used in this study.

GEO Dataset	No. of Samples			Platform ID	References
	Normal	CRC	Total		
GSE44861	55	56	111	GPL3921	[25]
GSE20916	44	46	90	GPL570	[26]
GSE113513	14	14	28	GPL15207	[27]

## Data Availability

Availability of data and materials: GEO datasets used in this study can be obtained from the GEO database (https://www.ncbi.nlm.nih.gov/geo/, accessed on 5 January 2021).

## Supplementary Materials

The following supporting information can be downloaded at https://www.mdpi.com/article/10.3390/biology11030365/s1: Table S1. The experimental results from different models and performance comparison of all methods across training and test datasets; Table S2. List of identified differentially expressed genes and their respective *p*-values, and the fold changes; Table S3. A list of the identified genes and their miRNA associations.

## Abbreviations

AUROC	Area under the receiver operating characteristic curve
CRC	Colon cancer
DEGs	Differential expressed genes
GEO	Gene Expression Omnibus
GO	Gene ontology
miRNA	microRNA
MDI	Mean decrease in impurity
RF	Random forest
