# Supplementary material for: Machine Learning-Based Identification of Colon Cancer Candidate Diagnostics Genes
Source: Biology (Basel). 2022 Feb 25;11(3):365. doi: 10.3390/biology11030365 (PMC8944988; doi:10.3390/biology11030365)
Supplement: Supplementary file 1 [file biology-11-00365-s001.zip › biology-1537026-supplementary/Table S1.pdf]

Table S1: The experimental results of method for different models and performance comparison of the all the methods across training and test datasets.

Classifier	Train Data	Test Data	*Using bootstrap method with 100 iterations*							*Using Cross validation (kfold=5)*	*Using LOOCV*
			**Precision**	**Recall**	**Specificity**	**Sensitivity**	**F1**	**AUROC**	**Accuracy**	**Accuracy**	**Accuracy**
Logistic Regression	GSE44861	GSE20916	0.667	1	0.477	1	0.8	0.991	0.744	0.80	0.84
		**GSE113513**	**1**	**0.929**	**1**	**0.929**	**0.963**	**1**	**0.964**	0.80	0.84
	GSE20916	**GSE44861**	**0.907**	**0.875**	**0.909**	**0.875**	**0.891**	**0.944**	**0.892**	1.0	1.0
		GSE113513	1	1	1	1	1	1	1.0	1.0	1.0
	GSE113513	GSE44861	0.505	1	0	1	0.671	0.948	0.505	1.0	1.0
		GSE20916	0.511	1	0	1	0.676	0.994	0.511	1.0	1.0
Random Forest	GSE44861	**GSE20916**	**0.987**	**0.979**	**0.986**	**0.979**	**0.983**	**0.999**	**0.982**	0.86	0.90
		GSE113513	0.5	1	0	1	0.667	0.583	0.5	0.87	0.90
	GSE20916	**GSE44861**	**0.809**	**0.906**	**0.78**	**0.906**	**0.854**	**0.914**	**0.844**	1.0	0.98
		GSE113513	0.17	0.0136	1	0.0136	0.0249	0.792	0.507	0.99	1.0
	GSE113513	GSE44861	0.504	1	0	1	0.671	0.505	0.505	1.0	1.0
		GSE20916	0.511	1	0	1	0.676	0.507	0.511	1.0	1.0
ExtraTrees Classifier	GSE44861	**GSE20916**	**0.857**	**0.906**	**0.845**	**0.906**	**0.88**	**0.934**	**0.876**	0.86	0.87
		GSE113513	0.02	0.001	1	0.001	0.003	0.914	0.501	0.86	0.89
	GSE20916	**GSE44861**	**0.86**	**0.899**	**0.85**	**0.899**	**0.878**	**0.934**	**0.875**	1.0	1.0
		GSE113513	0.05	0.008	1	0.008	0.013	0.916	0.504	1.0	1.0
	GSE113513	**GSE44861**	**0.858**	**0.908**	**0.846**	**0.908**	**0.882**	**0.934**	**0.877**	1.0	1.0
		GSE20916	0.01	0.001	1	0.001	0.001	0.914	0.5	1.0	1.0
NB Classifier	GSE44861	GSE20916	0.541	1	0.114	1	0.702	0.625	0.567	0.84	0.86
		GSE113513	0.5	1	0	1	0.667	0.5	0.5	0.83	0.87

	GSE20916	**GSE44861**	**0.895**	**0.911**	**0.891**	**0.911**	**0.903**	**0.91**	**0.901**	0.98	0.96
		GSE113513	0.5	1	0	1	0.667	0.5	0.5	0.98	0.98
	GSE113513	GSE44861	0.505	1	0	1	0.671	0.5	0.505	1.0	1.0
		GSE20916	0.511	1	0	1	0.676	0.5	0.511	1.0	1.0
XGBoost Classifier	GSE44861	**GSE20916**	**0.978**	**0.957**	**0.977**	**0.957**	**0.967**	**0.993**	**0.967**	0.83	0.85
		GSE113513	0.6111	0.786	0.5	0.786	0.688	0.638	0.643	0.83	0.85
	GSE20916	GSE44861	0.554	1	0.182	1	0.713	0.851	0.595	0.98	0.97
		GSE113513	0.5	1	0	1	0.667	0.5	0.5	0.97	0.97
	GSE113513	GSE44861	0	0	1	0	0	0.5	0.495	0.97	0.96
		GSE20916	0	0	1	0	0	0.5	0.489	0.97	0.96
Adaboost Classifier	GSE44861	**GSE20916**	**0.821**	**1**	**0.773**	**1**	**0.902**	**0.999**	**0.889**	0.86	0.84
		GSE113513	0.765	0.929	0.714	0.929	0.839	0.811	0.821	0.86	0.84
	GSE20916	GSE44861	0.83	0.696	0.855	0.696	0.757	0.775	0.775	0.97	0.98
		GSE113513	0	0	1	0	0	0.5	0.5	0.97	0.98
	GSE113513	GSE44861	0.505	1	0	1	0.671	0.5	0.505	0.90	0.93
		GSE20916	0.511	1	0	1	0.676	0.5	0.511	0.90	0.93
